# Coinfection of High-Risk Human Papillomavirus and Lower Genital Tract Pathogens in the Development of High-Grade Cervical Lesions

**DOI:** 10.1155/2020/7640758

**Published:** 2020-08-30

**Authors:** Hui Zhong, Yao Tong, Haifeng Lin, Xiaodan Mao, Binhua Dong, Zhihui Wu, Huiyu Chen, Pengming Sun

**Affiliations:** ^1^Clinical Laboratory, Fujian Maternity and Child Health Hospital, Affiliated Hospital of Fujian Medical University, Fuzhou 350001, China; ^2^Laboratory of Gynecologic Oncology, Fujian Maternity and Child Health Hospital, Affiliated Hospital of Fujian Medical University, Fuzhou 350001, China

## Abstract

**Purpose:**

This study investigated the infection status and relationship between other common lower genital tract infectious pathogens and high-risk human papillomavirus (HR-HPV) in the high-grade cervical lesions.

**Methods:**

Overall, 882 patients were enrolled in this retrospective study, of which 339 patients (≥HSIL group) were confirmed with high-grade squamous intraepithelial lesions (HSIL) or cervical squamous cell carcinoma (SCC), while 543 patients (≤LSIL group) were diagnosed with low-grade squamous intraepithelial lesions (LSIL) or normal cervical pathology diagnosis. Cervical swab specimens were tested for HPV, pathogenic bacteria (PB), *U. urealyticum* (UU), *M. hominis* (MH), *and C. trachomatis* (CT) in both groups.

**Results:**

The infection rates of HR-HPV, PB, UU (at high density), and CT were higher in the ≥HSIL group than in the ≤LSIL group (*P* < 0.001); however, higher infection rates with MH were not observed (*P* > 0.05). PB, UU, and CT were associated with HR-HPV infection (*P* < 0.001). The PB and UU infection rates in the ≥HSIL group were significantly different from those in the ≤LSIL group, regardless of whether there was an HR-HPV infection at the same time (*P* < 0.05). However, this was not the case for the CT (*P* > 0.05). Furthermore, 259 pathogenic bacterial strains were detected in 882 cases. The difference in the distribution of pathogenic bacterial flora in the different grades of cervical lesions had no statistical significance, which was prioritized over *Escherichia coli* (*P* > 0.05).

**Conclusion:**

PB, UU, and CT infection is associated with susceptibility to HR-HPV, HR-HPV coinfection with these pathogens might increase the risk of high-grade cervical lesions, and PB and UU might be independent risk factors for cervical lesions.

## 1. Introduction

Cervical squamous cell carcinoma (SCC) is one of the most common malignant tumors in gynecology [1]. High-grade squamous intraepithelial lesions (HSIL) are a precancerous lesion closely related to SCC. Biological and epidemiological studies have confirmed that persistent infection of high-risk human papillomavirus (HR-HPV) is the leading cause of cervical precancerous lesions and SCC [2]. Most HPV infections are transient and cleared by host immune responses, while only a small percentage of infections persist and progress to HSIL and invasive SCC [3], which suggests that there are other synergistic factors in the development of cervical epithelial cells into malignant lesions after HPV infection.

Recent studies have found that certain HPV types and HPV viral loads may be related to HPV infection persistence and cancer progression [4]. In addition, several possible risk factors lead to the development of cervical lesions, such as first sexual intercourse at an early age [5], smoking [6], multiple sexual partners [7], impaired immune function [8], and hormonal contraceptives [9], and more importantly, studies have found that patients with lower genital tract infection (LGTI) other than HPV have an increased risk of developing SCC [10].

The incidence of lower genital tract infections has been increasing in recent years. An epidemiological survey of women in Beijing, China, showed that 11.4% of women had LGTIs [11]. Previous studies have found that genital pathogen infection may affect the susceptibility and clearance of HPV infection, and chronic inflammation caused by nonspecific genital infection is associated with SCC [12–15]. However, there is no consensus on the role of lower genital tract infection in high-grade cervical lesions.

The aim of this study was to evaluate the prevalence of HR-HPV and other lower genital tract pathogens in different grades of cervical lesions in Fujian, China, and investigate the correlations of coinfection of HR-HPV and lower genital tract pathogens on the risk of high-grade cervical disease.

## 2. Materials and Methods

### 2.1. Study Population

**Figure 1:**
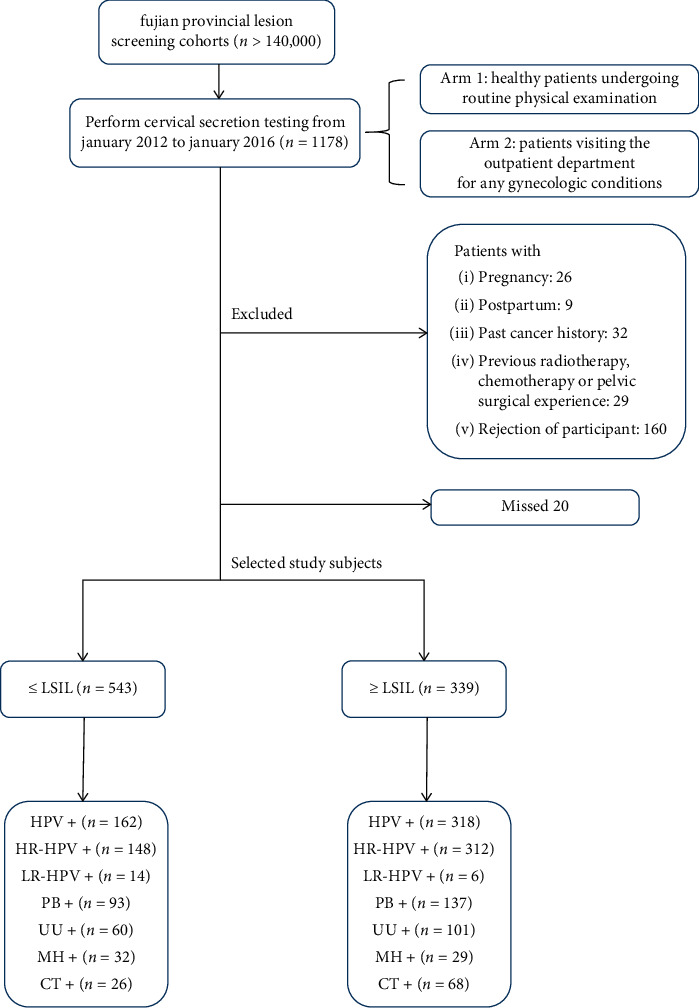
Flow chart of the patients included. ≤LSIL: normal or low-grade squamous intraepithelial lesion; ≥HSIL: high-grade squamous intraepithelial lesion or cervical squamous cell carcinoma; HPV: human papillomavirus; HR-HPV: high-risk human papillomavirus; LR-HPV: low-risk human papillomavirus; PB: pathogenic bacteria; UU: *U. urealyticum;* MH: *M. hominis;* CT: *C. trachomatis*.

The study population was from Fujian provincial lesion screening cohorts (*n* > 140,000) and had undergone cervical secretion testing, which involved 2 cohorts, one consisting of healthy patients undergoing routine physical examination and the other consisting of patients visiting the outpatient clinic for any gynecologic conditions. All women received cervical secretion testing between January 2012 and January 2016 (*n* = 1178). The participants were required to meet the following criteria: (1) women in nonmenstrual period; (2) nongestational period, nonlactation period; (3) no acute and chronic illness; (4) no hysterectomy history; (5) no vaginal washing three days before sampling; (6) no use of contraceptives and other vaginal drugs; (7) no previous experience of radiotherapy, chemotherapy, or surgery. The study was approved by the Ethics Committee of Fujian Maternity and Child Health Hospital, Affiliated Hospital of Fujian Medical University (2016-002) and obtained the exemption of informed consent. The study flowchart is shown in Figure 1.

### 2.2. Specimen Collection

Gynecologists performed routine gynecological examinations. Cervical cytology samples were collected from all subjects using plastic brushes, which were then immersed in a preservation solution for HPV DNA testing, and stored at −20°C. At the same time, cervical samples were collected using sterile cotton swabs for the detection of pathogens *U. urealyticum* (UU), *M. hominis* (MH), and *C. trachomatis* (CT).

### 2.3. HPV Detection and Typing

Cervical exfoliated cells for polymerase chain reaction-reverse dot blot HPV genotyping (Yaneng Biotech, Shenzhen, China) were obtained from all subjects. Twenty-three HPV genotypes were detected, including 18 HR-HPV (16, 18, 31, 33, 35, 39, 45, 51, 52, 53, 56, 58, 59, 66, 68, 73, 82, and 83) and five low-risk HPV (6, 11, 42, 43, and 81) genotypes. All steps were performed in accordance with the manufacturer's instructions, and HPV DNA was amplified in a thermal cycler that included denaturation, annealing, extension, and hybridization. Finally, the blue spots on the strips, which were fixed with the 23 different HPV probes, were identified as positive.

### 2.4. Bacterial Testing

After obtaining the cervical swabs, they were sent to the laboratory immediately. Culture was performed on blood agar plates, chocolate agar plates, and Sabouraud's dextrose agar plates (Beiruite, Zhengzhou, China) and incubated for up to 48 h in 5–10% CO_2_ at 37°C. Individual colonies with large numbers of bacteria were selected, and clinical isolates were identified using standard microbiological methods. VITEK 2 Gram-positive-GP ID and Gram-negative-GN ID cards (BioMérieux, Balmes-les-Grottes, France), based on colorimetric detection, were used for the identification of bacteria according to the manufacturer's instructions. *Escherichia coli* ATCC 25922, *Pseudomonas aeruginosa* ATCC 27853, *Staphylococcus aureus* ATCC 25923, and *Enterobacter cloacae* ATCC 700323 strains were used as controls.

### 2.5. UU/MH/CT Testing

Cervical specimens were collected for UU, MH, and CT testing. UU and MH were isolated by Mycoplasma IST 2 (BioMérieux), using the liquid culture method. Diagnostic criteria were based on the presence or absence of MH and UU and an estimate of concentration variation (cut-off 104-color-changing units-CCU/mL). CT was detected using an immunochromatography assay (Clearview; Unipath Ltd., Bedford, UK).

### 2.6. Statistical Analysis

Data were analyzed using SPSS 22.0 (IBM Corp., Armonk, NY, USA). The associations between other lower genital tract pathogens and HR-HPV infection were determined using chi-square tests. To compare the LGTIs positive rates based on pathological diagnosis, chi-square tests or Fisher exact tests were performed. Multivariate logistic regression analysis was used to assess the simultaneous effect of more than one variable on the risk of high-grade cervical lesions and to identify possible confounding factors. Data are reported as numbers (%) or odds ratios (OR) with the corresponding 95% CI. *P* < 0.05 were considered statistically significant.

## 3. Results

### 3.1. Prevalence of Pathogens in Different Cervical Lesions

A total of 458 (51.9%) women were with normal cervical pathology, and 424 (48.1%) women exhibited the following abnormal pathology: 85 LSIL (9.6%), 139 HSIL (15.8%), and 200 SCC (22.7%) (Figure 1). Women with normal pathology and LSIL were significantly younger (43.13 ± 10.321 years) than women who had HSIL and SCC (45.79 ± 8.791 years) (*P* < 0.001).

**Figure 2:**
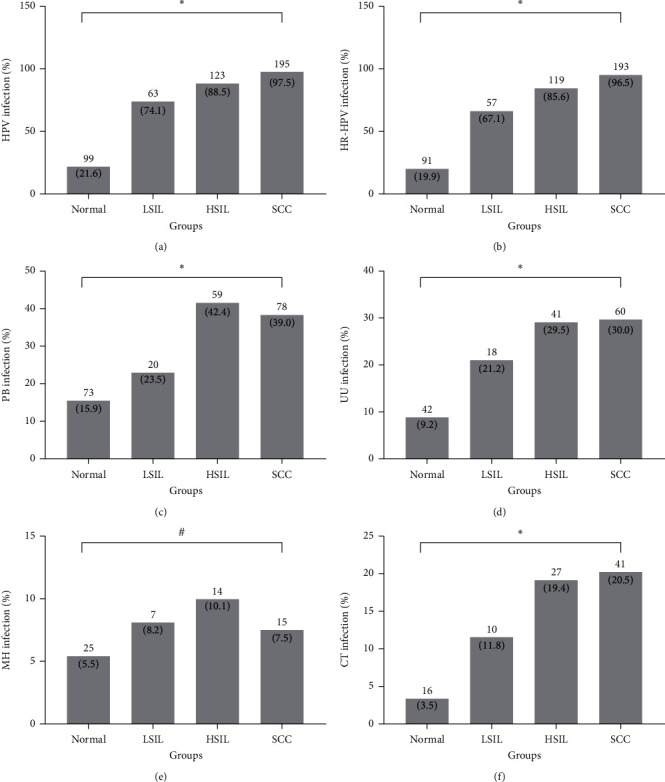
Infection rate of lower genital tract pathogens in different groups. LSIL: low‐grade squamous intraepithelial lesion; HSIL: high‐grade squamous intraepithelial lesion; HPV: human papillomavirus; HR-HPV: high‐risk human papillomavirus; SCC: squamous cell carcinoma; PB: pathogenic bacteria; UU: *U. urealyticum*; MH: *M. hominis*; CT: *C. trachomatis*. ^∗^Comparison between groups (*P* > 0.001) (chi-square analysis). #Comparison between groups (*P* > 0.05) (chi-square analysis).

The highest rate of prevalence was attributed to HPV infection (54.4%, 480/882), with HR-HPV infection (52.2%, 460/882). This was followed by PB (26.0%, 230/882), UU (18.3%, 161/882), CT (10.7%, 94/882), and MH (6.9%, 61/882). As expected, as cervical pathology became more severe, the positive rate for HR-HPV significantly increased (*P* < 0.001), as shown in Figure 2. The positive rates for PB, UU, and CT were significantly different according to the cervical pathology results (*P* < 0.001, *P* < 0.001, and *P* < 0.001, respectively), as shown in Figure 2. No significant differences were observed in low-risk HPV, LR-HPV (*P* = 0.433) and MH (*P* = 0.433, *P* = 0.130, respectively). Multivariate logistic regression analysis suggested that menopausal status, HPV, HR-HPV, PB, UU, and CT were independent risk factors for high-grade cervical lesions (Table 1).

**Figure 3:**
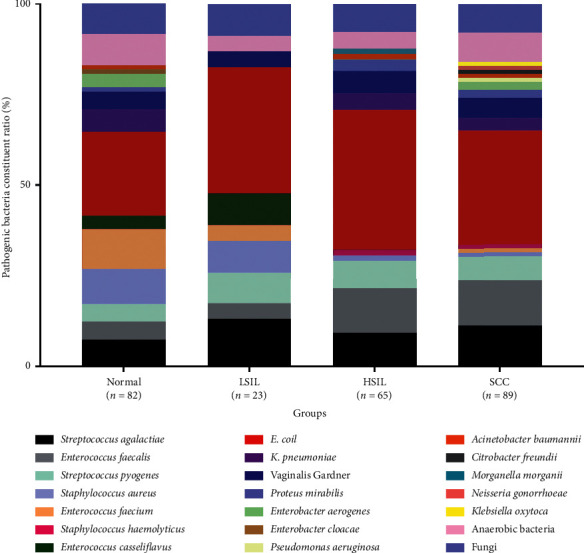
Distribution and constituent ratio of pathogenic bacteria in different cervical lesions groups. ≤LSIL: normal or low-grade squamous intraepithelial lesion; ≥HSIL: high‐grade squamous intraepithelial lesion or cervical cancer; SCC: squamous cell carcinoma.

In our study, a total of 259 PB strains were detected, and the distribution is shown in Figure 3. Among 458 samples in the normal group, 73 (15.9%) women presented with PB infection; of the 85 samples in the LSIL group, 20 (23.5%) were infected with PB; of the 139 samples in the HSIL group, 59 (42.4%) were infected with PB; and of the 200 samples in SCC group, 78 (39.0%) women presented with PB infection. 12 women were infected with two cases of bacteria in ≤LSIL group, and 17 had two cases of bacteria in ≥HSIL group. The difference in the distribution of PB flora in the normal, LSIL, HSIL, and SCC groups was not significant, which was prioritized over *Escherichia coli (E. coli)* (*P* > 0.05, Figure 3).

### 3.2. Coinfection with HR-HPV and Other Pathogens

Infection with other pathogens was frequently detected in HPV positive women. In fact, PB were more likely to be diagnosed in HR-HPV positive women than women who were HR-HPV negative (35.4% [163/460] vs. 15.9% [67/422]), and a significant association between presence of HR-HPV and PB infection was observed (*P* < 0.001). Similarly, UU was detected in 27.2% (125/460) of HR-HPV positive patients and CT in 16.5% (76/460) of HR-HPV positive patients. UU and CT were more likely to be diagnosed in HR-HPV positive women than in those who were HR-HPV negative (*P* < 0.001, *P* < 0.001, respectively). The prevalence of MH was 6.7% (31/460) among HR-HPV positive women and 7.1% (30/422) among HR-HPV negative women. Statistical analysis did not reveal any association between the presence of HR-HPV and MH (*P* > 0.05, Table 2).

### 3.3. Risk of HR-HPV Coinfection with Other Lower Genital Tract Pathogens for High-Grade Cervical Lesions

Table 2 shows the risk of coinfection of HR-HPV with PB, UU, CT, and different grades of cervical lesions. Coinfection of HR-HPV and pathogens showed a higher increased risk for ≥HSIL (OR: 2.250, 95% CI: 1.423–3.557, *P* < 0.001). Coinfection of HR-HPV and UU increased risk of ≥HSIL (OR: 1.791, 95% CI: 1.096–2.926, *P*=0.020). Coinfections of CT plus HR-HPV increased the risk of ≥HSIL (OR: 3.070, 95% CI: 1.540–6.120, *P*=0.001). No similar trends were observed in MH.

## 4. Discussion

As expected, we found a significant association between PB, UU, CT, and HR-HPV infection, and HR-HPV coinfection with these pathogens increased the risk of high-grade cervical disease. Among them, PB or UU infections may contribute to development of high-grade cervical lesions as an independent risk factor, regardless of whether there is HR-HPV infection. In contrast, we did not find a correlation between MH and HR-HPV or high-grade cervical lesions. In addition, in this study, the distribution of PB flora was also not statistically different between different grades of cervical lesions group.

A survey of cervical HPV prevalence in China showed that, among the cervical intraepithelial neoplasia (CIN)2+ patients, the HPV infection rate was 84.97% [16], which was slightly lower than the infection rate of HPV in our ≥HSIL group (93.8%). Local inflammation caused by lower reproductive tract infection leads to local metaplasia, which may increase the chance of HR-HPV infection and HR-HPV viral load. In this study, we found that women infected with pathogens had an increased risk of coinfection with HR-HPV, and a similar trend was found in UU and CT. This supports the hypothesis that these pathogens are closely related to persistent HR-HPV infection.

The healthy cervical bacterial community usually consists of *Lactobacillus* spp. It contains about 90% of the cervical bacterial flora and serves as the first line of defense against pathogenic bacteria [17]. More and more evidence shows that the microflora environment of the lower reproductive tract may be related to HPV infection, persistent infection, local mucosal immunity, and cervical lesions [18]. Vaginal flora imbalance, especially lactic acid bacteria, may lead to cervical cytology abnormalities, leading to an increased risk of cervical cancer precancerous lesions and cervical cancer [19–21]. In our study, the lower genital pathogen was the most common non-HPV pathogen, HR-HPV infection is prone to coinfection with pathogens, and coinfection with HPV increases the risk of high-grade cervical diseases, which was essentially in agreement with another study [22]. In addition, our study did not find that the increase in vaginal microbiota diversity is associated with the occurrence of cervical lesions and may be related to the different detection of pathogenic microorganisms (for example, conventional microscopy and/or culture methods and PCR-based methods).

CT is one of the most widely studied sexually transmitted pathogens. Most epidemiological studies have shown that *Chlamydia trachomatis* infection rates are higher in patients with cervical cancer [23–26]. Discacciati MG et al. found that CT induces MMP-9/RECK imbalance during cervical inflammation, and this imbalance plays an important role in HPV-mediated cervical cancer [27]. CT infection can also reduce the host's ability to resolve HPV infection by increasing free radical production and reducing host cell-mediated immunity, resulting in persistent HPV infection [28]. CT may play a specific role in the history of cervical lesions through the above mechanisms. This is consistent with our findings. In our study, CT and HR-HPV coinfection showed a 3.288-fold increase in the risk of high-grade cervical lesions, but there was no significant difference in patients without HR-HPV infection. Therefore, we speculate that CT and HR-HPV act synergistically as one of the risk factors for high-grade cervical lesions. Our findings emphasize the need for further studies to assess the actual impact of coinfection on the risk of different grades of cervical lesions.

In addition, Lukic et al. [29] found that the higher the level of cervical lesions, the higher the UU infection rate (35% for LSIL and 45% for HSIL). Xiao et al. [12] found that the significant combination of HPV infection and UU can strengthen the development of the disease and lead to the pathogenesis of cervical cancer. All the above studies are consistent with our study, UU infection was significantly associated with HR-HPV infection, and UU might be independent risk factor for cervical lesions. Further research is needed to identify the problem and the potential mechanisms associated with these pathogens.

In our research population, the prevalence of mixed infections deserves the attention of the public health services. Our study further demonstrates the efficacy of coinfection with HR-HPV and other lower genital pathogens, supporting the high coinfection rate of pathogens, CT, UU, and HR-HPV, and their possible risk in high-grade cervical lesions. Whether it is HR-HPV, pathogens, CT, or UU, the mode of transmission is closely related to sexual intercourse. Therefore, advocating healthy sexual behavior is essential for the prevention and treatment of cervical lesions. It is suggested that, in the screening of clinical SCC and precancerous lesions, in addition to detecting HR-HPV, vaginal microecological detection should be carried out, attention should be paid to the balance of pathogens in the lower genital tract pathogens, and the coinfection of pathogens, CT, UU, and HR-HPV should be screened to block its ability to affect the host's ability to clear HPV infection to determine appropriate diagnostic and therapeutic methods.

The advantages of our research include a relatively large number of patients, and the percentage of data missing in patients who participated in the study and met the inclusion and exclusion criteria was small. It is worth noting that our results are based on a summary analysis of rough epidemiological data, with moderate heterogeneity in the study, and without considering risk factors associated with sexual behavior in the study population. In addition, our study is mainly a cross-sectional study, lacking longitudinal observation of HR-HPV and other cervical pathogen infection processes and therefore it is difficult to study the relationship between persistent HR-HPV infection, genital pathogen infections, and cervical lesions. Future prospective cohort studies need to explore their relationship from a vertical perspective.

In conclusion, this study provided an opportunity to detect HPV simultaneous detection of pathogens and determine the rate of coinfection between HR-HPV and other lower genital tract pathogens in different grades of cervical lesions. Our results reinforce the hypothesis that some lower genital tract pathogens may be associated with HR-HPV infection and may be a risk factor for high-grade cervical lesions. This study was considered critical to further elucidate the pathogenesis of high-grade cervical disease and to improve early screening and intervention strategies.

## Figures and Tables

**Table 1:** Correlation between lower genital tract pathogens and cervical lesions.

Variable	≤LSIL (*n* = 543)	≥HSIL (*n* = 339)	*P* value	OR_adjust_ (95% CI)	*P* value_adjust_
Age	43.13 ± 10.321	45.79 ± 8.791	0.001^a^	—	—
Age					
≥50	92 (16.9)	98 (28.9)	0.001^b^	0.600	0.173
<50	451 (83.1)	241 (71.1)		(0.288–1.251)	
Menopause					
Yes	121 (22.3)	146 (43.1)	0.001^b^	3.503	0.001
No	422 (77.7)	193 (56.9)		(1.797–6.826)	
HPV					
Positive	162 (29.8)	318 (93.8)	0.018^b^	4.786	0.006
Negative	381 (70.2)	21 (6.2)		(1.558–14.709)	
HR-HPV					
Positive	148 (27.3)	312 (92.0)	0.001^b^	5.755	0.001
Negative	395 (72.7)	27 (8.0)		(2.033–16.294)	
LR-HPV					
Positive	14 (2.6)	6 (1.8)	0.433^b^	—	—
Negative	529 (97.4)	333 (98.2)			
Pathogenic bacteria					
Positive	93 ( 17.1)	137 (40.4)	0.001^b^	2.277	<0.001
Negative	450 (82.9)	202 (59.6)		(1.510–3.434)	
*U. urealyticum*					
Positive	60 (11.0)	101 (29.8)	0.001^b^	2.302	<0.001
Negative	483 (89.0)	238 (70.2)		(1.463–3.621)	
*M. hominis*					
Positive	32 (5.9)	29 (8.6)	0.130^b^	—	—
Negative	511 (94.1)	310 (91.4)			
*C. trachomatis*					
Positive	26 (4.8)	68 (20.1)	0.001^b^	3.066	<0.001
Negative	517 (95.2)	271 (79.9)		(1.672–5.623)	

^a^ *P* value for Student's *t*-test; ^b^*P* value for *χ*^2^ test; —: not applicable; adjust: after adjustment for age, menopausal status, and other pathogens. ≤LSIL: normal or low-grade squamous intraepithelial lesion; ≥HSIL: high-grade squamous intraepithelial lesion or cervical squamous cell carcinoma; HR-HPV: high-risk human papillomavirus; LR-HPV: low-risk human papillomavirus.

**Table 2:** Rates of HR-HPV and other pathogens coinfections and risk of the coinfections in high-grade cervical lesions.

Coinfection status		Overall	≤LSIL	≥HSIL	*P* value^a^	*P* value^b^	OR (95% CI)	OR_adjust_ (95% CI)	*P*_adjust_
HR-HPV (+)	PB (−)	297	113 (38.0)	184 (62.0)	0.001^*∗*^	0.001^*∗*^	1	1	0.001^*∗*^
	PB (+)	163	35 (21.5)	128 (78.5)			2.246 (1.445–3.491)	2.250 (1.423–3.557)	
HR-HPV (−)	PB (−)	355	337 (94.9)	18 (5.1)	0.024^*∗*^		1	1	0.023^*∗*^
	PB (+)	67	58 (86.6)	9 (13.4)			2.905 (1.245–6.779)	2.779 (1.154–6.689)	
HR-HPV (+)	UU (−)	335	118 (35.2)	217 (65.8)	0.022^*∗*^	0.001^*∗*^	1	1	0.020^*∗*^
	UU (+)	125	30 (24.0)	95 (76.0)			1.722 (1.079–2.749)	1.791 (1.096–2.926)	
HR-HPV (−)	UU (−)	386	365 (94.6)	21 (5.4)	0.020^*∗*^		1	1	0.021^*∗*^
	UU (+)	36	30 (83.3)	6 (6.7)			3.476 (1.304–9.269)	4.163 (1.243–13.941)	
HR-HPV (+)	MH (−)	429	143 (14.5)	286 (85.5)	0.048^*∗*^	0.829	1	1	0.202
	MH (+)	31	5 (35.7)	26 (64.3)			2.600 (0.978–6.913)	1.930 (0.703–5.298)	
HR-HPV (−)	MH (−)	392	368 (93.9)	24 (6.1)	0.427		1	1	0.461
	MH (+)	30	27 (90.0)	3 (10.0)			1.704 (0.482–6.020)	0.551 (0.113–2.683)	
HR-HPV (+)	CT (−)	384	137 (35.7)	247 (64.3)	0.001^*∗*^	0.001^*∗*^	1	1	0.001^*∗*^
	CT (+)	76	11 (14.5)	65 (85.5)			3.278 (1.673–6.419)	3.070 (1.540–6.120)	
HR-HPV (−)	CT (−)	404	380 (94.1)	24 (5.9)	0.100		1	1	0.096
	CT (+)	18	15 (83.3)	3 (6.7)			3.167 (0.857–11.694)	3.190 (0.815–12.494)	

Values are presented as number (%). ^a^Compared in different lower genital tract pathogens infection states. ^b^Compared in different HR-HPV infection states. _adjust_: after adjustment for age and menopausal status. ^*∗*^*P* < 0.05. ≤LSIL: normal or low-grade squamous intraepithelial lesion; ≥HSIL: high-grade squamous intraepithelial lesion or cervical squamous cell carcinoma; HR-HPV: high-risk human papillomavirus; OR: odds ratio.

## Data Availability

The data used to support the findings of this study are available from the corresponding author upon request.
